# Narrative portraits: affirmative approaches to understanding learning disability in the everyday

**DOI:** 10.3389/fsoc.2025.1560701

**Published:** 2025-04-03

**Authors:** Tom Ryan

**Affiliations:** School of Education, University of Sheffield, Sheffield, United Kingdom

**Keywords:** learning disability, narrative portraits, everyday sociology, qualitative research, narrative inquiry

## Abstract

Narrative portraits provide an opportunity to uncover new affirmative understandings of disability and family through the focus on lived experiences. This article will explore how a critical disability studies lens helps us understand narrative approaches and the crip potentials of narrative portraits. Considering the ‘joy deficit’ within disability research this paper highlights the disruptive potential narrative portraits bring to family sociology and disability studies. This paper presents a narrative portrait as a case study, taken from research carried out with 14 siblings of people with learning disabilities from the UK. This is used to explore how siblings of people with learning disabilities understand disability in the everyday with a focus on the affirmative and disruptive counter-narrative nature of the portrait. Through this, the potential for counter-narratives within this methodology will be made clear with the unique nature of sibling relationships central to this. Narrative inquiry can challenge dominant deficit understandings of disability through narrative repair. Narrative portraits take this further through the focus on participants’ words in longer extracts allowing their viewpoints to be centred. This approach lends itself to studies of the everyday through the space afforded for deeper, nuanced accounts of life. The approach crips more classic narrative research methods through challenging normative understandings of the researcher’s role in favour of a more participant-centred approach to analysis. In doing so, there is potential to imagine a more inclusive scholarship. When addressed through a disability lens, narrative portraiture uncovers lived experiences of disability, how disability is navigated in families, and how siblings negotiate disability in their relationships allowing the nuances of everyday experiences of disability to arise.

## Introduction

Narrative portraits offer the potential to generate new affirmative understandings of disability and family through the focus on lived experiences. By centring the participants’ own words, with a particular consideration to context, portraiture allows for counter-narratives to emerge clearly. Drawing on narrative inquiry, portraiture sees research data presented in a way that gives the audience insights into participants’ narratives through extended sections of their own words that are brought together by the researcher with the aim of both respecting their narrative and addressing research aims (Rodríguez-Dorans, 2022). This paper draws on one narrative portrait as a case study to make clear the affirmative potential of narrative portraiture in understanding learning disability in the everyday.

Sibling relationships are nuanced and complex (Davies, 2023); they can be characterised by both loving and caring feelings as well as more frustrating and at times conflictual ones (Davies, 2015). Disabled people are not always afforded this nuance in research around siblinghood and disability, which leads to commonplace deficit narratives being reproduced through a focus on non-disabled sibling outcomes (see Meltzer and Kramer, 2016 and Ryan, 2024 for overviews). When considering the need for affirmative learning disability and siblinghood research, Shuster and Westbrook’s (2022) conceptualisation of ‘joy deficit’ offers a clear argument for the importance of challenging commonplace deficit narratives in research around marginalized communities. They argue that due to the focus on social harms in much of social science, research outputs inevitably contribute to understandings of marginalized communities that are centred around harms and, as a result, deficit. This then influences understandings of lived experiences socially and culturally leading to deficit narratives being commonplace within society. The authors call for more research that acknowledges joy, an argument that has been applied to disability studies research specifically (Sunderland et al., 2009) with this paper working to contribute to understandings of learning disability and family that allow joy to be a central part of relationships. Narrative portraits are one way to achieve this through their potential for ‘more detailed, descriptive, and richer narratives that reveal more of the identity and interests of participants and researchers’ (Smyth and McInerney, 2013: 4).

Drawing on a wider research project exploring the experiences of siblings of people with learning disabilities, this article will make clear the potential of narrative portraiture in presenting counter-narratives that challenge dominant understandings of siblinghood and disability. This will be achieved through an introduction to debates within siblinghood and learning disability research, highlighting the importance of counter-narratives. Following this, the concept of narrative repair and the affirmative potential this brings (Lindemann, 2001) will be discussed. Narrative portraiture will then be explored, with reference to analysing and creating portraits, which leads to the methodology of this paper. Toby’s narrative portrait is then presented, which explores his relationship with his sister Beth through their love of playing video games together and how this has changed as they have grown older and Toby has moved away. The portrait is analysed following the Labovian-influenced (Labov and Waletzky, 1997) approach advocated by Rodríguez-Dorans (2022) with particular attention on the affirmative aspects of Toby’s narrative.

### Thinking about siblinghood in the everyday

Being a sibling is a unique relationship, often cited as one of the longest people will have in their lifetime (Allan, 1979), navigating a number of changes over this time. Siblings can be a great support in times that are difficult and in other moments frustrating and conflictual. We tend to understand these feelings, even the more aggressive ones, as to be expected from siblings, especially in childhood (Davies, 2023). Davies (2015) refers to the ‘emotional tightrope’ that characterises sibling relationships as almost simultaneously loving and more visceral. These understandings are rooted in the mundane everyday experiences of growing up together, with this leading to moments of closeness and love but also sometimes conflict and frustration. This is seen in Morgan’s (2011) conceptualisation of ‘family practices’ which is rooted within the everyday ‘both in the sense of those life events which are experienced by a significant proportion of any population (partnering, parenting, sickness, bereavement) and, equally, those activities which seem unremarkable’ (Morgan, 2011: 6). Punch (2008) reflects on the nature of sibling relationships drawing on Goffman’s (1969) dramaturgical metaphor to place siblinghood as a ‘backstage’ relationship leading to siblings engaging ‘in a backstage informal presentation of self without fear of the consequences of putting on an unpolished performance’ (ibid: 341). Furthermore, Davies (2023) refers to ‘living alongside’ to capture the everyday-ness of sibling relationships with a particular focus on the heightened proximity and intimacy this brings. This context highlights the importance of nuance in how we analyse sibling relationships, with there being a need for recognition of the ups and downs that are to be expected of growing up alongside one another.

When considering siblinghood and disability, it is important to acknowledge how research exploring this has often reproduced deficit understandings. Meltzer (2018) reflects on this, arguing ‘sibling disability research has traditionally defined the relationships between siblings where one has a disability by what they are not—that is, when compared to the normative view of relationships between siblings where neither have a disability, sibling relationships that include a person with a disability have traditionally been found wanting or damaged in comparison’ (Meltzer, 2018: 1228). This presentation often draws upon understandings of disability as deviation from the ‘ideal type’ (Garland-Thomson, 2007). This will be revisited further into the paper. In response to presentations of ‘wanting’ or ‘damaged’ sibling relationships, Meltzer (2018) argues for a more nuanced reading of disability and siblinghood. The importance of allowing for this nuance when exploring siblinghood and learning disability has been highlighted in previous research. For example, in the study by Cebula et al. (2024) about the experiences of siblings of people with Williams syndrome, they note the closeness and warmth of the relationships and call for more research that approaches sibling relationships in a more holistic manner. Similarly, research by Stalker and Connors (2004) with siblings of people with learning disabilities challenges pathological narratives in favour of reflections of the everyday that echo wider family and sibling research. Moran-Morbey et al. (2024) further highlight the importance of recognising the structural factors at play, and how these must be part of how we understand the experiences of siblings to ensure we move away from individualising, pathological narratives of disability being reproduced. These examples present an articulation of siblinghood that is more in line with wider family sociology understandings as nuanced and complex.

### Narrative inquiry and disability studies

Narrative inquiry offers a path towards more holistic understandings of disability and siblinghood through focusing on longer periods and more in-depth data allowing for nuances to come through more clearly. For Wells (2011), narrative inquiry is interested in looking at language as opposed to ‘through it’. To achieve this, researchers must explore how a story unfolds over time, the performance of the narrative, narrative structure, its reception, and the cultural contexts in which it is based. Narratives can be understood in a number of ways, including “folk theories’, ‘frames’, ‘scripts’, ‘mental models’, ‘cultural models’, ‘discourse models’, ‘social models’, and ‘figured worlds” (Gee, 2005: 89). For Frank (2010), narratives are best understood as stories, something they argue researchers should embrace. Stories are a key part of how we create meaning (Thavakugathasalingam and Schwind, 2022); indeed, Gubrium and Holstein (2009) argue that narrative practices are how we bring meaning to experiences. This framing recognises that narratives can only be effective if they are understood by their audience. This is commonplace in narrative inquiry reflections; for example, Freeman (2007) notes how stories, whilst expressed by ‘a self’, are made with others through discussion. Wells (2011) builds on this stating that narratives are informed by context, audience, and culturally specific understandings amongst other things, something that must be recognised in analysis. This recognition of narratives as needing to be understood by their audiences is taken further by Sparkes and Smith (2011) who use the phrase ‘conventions of reportage’ to refer to the importance of drawing upon socially sanctioned viewpoints or risk people not understanding a narrative. They build on Medved and Brockmeier’s understanding of narrative as fitting within a ‘generalised and culturally established canon’ (Medved and Brockmeier, 2008: 469).

Within this framework, we begin to see the potential questions that can arise when an individual’s narrative does not coincide with this ‘culturally established canon’ (Medved and Brockmeier, 2008: 469) and the work that can go into attempting to fit within these boundaries. Where narratives are seen to create meaning, we see the power that narratives have to influence how disabled people are understood culturally, with examples such as the study by Grue (2016) on inspiration porn and Jarrett’s (2020) exploration of understandings of learning disability historically highlighting the damaging nature of popular narratives of disability. Sparkes and Smith (2011) go on to note the implications of not fitting it, noting that ‘stimulating narrative imagination’ works to ‘achieve solidarity and bond with others empathetically as fellow human beings’ (ibid: 369). From this, we can see how narrative links to understandings of marginalised experiences with the space afforded by a narrative approach to lived experience and commitment to the whole bringing with it the potential for counternarratives to be generated (Solórzano and Yosso, 2002), something that will be explored in further into the paper.

Narrative methodology brings with it questions of validity and narrative truth. Narrative portraiture is by no means immune from this discussion and leans into the role of ‘narrative truth’ in how we understand narratives as data. The question of validity in narrative research is one that is framed around ‘truths’, with Polkinghorne (2007) arguing ‘the language description given by participants of their experienced meaning is not a mirrored reflection of this meaning’ (ibid: 480). Spence (1982) refers to ‘narrative truths’, with Kalekin-Fishman (2016) building on this with the term ‘lived truths’. On the question of ‘truth’ in narrative research, Lieblich et al. (1998) argue “stories are usually constructed around a core of facts or life events, yet allow a wide periphery for the freedom of individuality and creativity in selection, addition to, emphasis on and interpretation of these ‘remembered facts”’ (ibid: 8). Returning to portraits specifically, and to the study by Rodríguez-Dorans (2022) which argues ‘portraits and the narratives that accompany them do not intend to present accurate realities, they are interpretations that aim to reflect people’s narrated experiences’ (Rodríguez-Dorans, 2022:80). This understanding of ‘narrated experience’ returns to conceptualisations of narrative as being central to how we create meaning (Hoffman, 1993; Thavakugathasalingam and Schwind, 2022), a sentiment captured in Garland-Thomson’s (2007) reflection ‘narratives do cultural work. They frame our understandings of raw, unorganised experience giving it coherent meaning and make it accessible to us through story’ (ibid: 122).

### Narrative repair and affirmative research

Counter-narrative research has its origins in critical race theory (Solórzano and Yosso, 2002) and works to challenge dominant majoritarian narratives through centring alternative perspectives (Klinge et al., 2020). Lived experiences, often from marginalised perspectives, are one of the key aspects of counternarratives (Delgado, 1984; Walker et al., 2020). For hooks (1994), counter-narratives are sites of resistances, as captured by Mohanty’s (1989) argument that ‘resistance lies in self-conscious engagement with dominant, normative discourses and representations and in the active creation of oppositional analytic and cultural spaces’ (ibid: 185). Nelson (2001) argues identities are constituted through narratives and in some instances subsequently damaged through this process. They argue counter-narratives act as a means to carry out ‘narrative-repair’. This process sees the damaged narrative replaced by more representative ones through the centring of counter-narratives.

This understanding places counter-narratives as radical spaces of resistance against dominant perceptions. Dominant understandings of learning disabilities often reinforce pathological, deficit-based understandings, and a counter-narrative approach provides an opportunity to challenge this. Leaning and Adderley (2016) refer to ‘problem-saturated narratives’ as commonplace in the understandings of disability. Garland-Thomson (2007) captures the role of narrative in how we understand disability culturally:

*‘Both our bodies and stories we tell about them are shaped to conform to a standard model of human form and function that is called normal in medical-scientific discourse, average in consumer capitalism and ordinary in colloquial parlance.’* (ibid: 114)

Garland-Thomson makes clear how narrative shapes understandings of disability, making the link between understandings of ‘ideal types’ and capitalism. The role of capitalism in shaping understandings of disability is well documented (see, e.g., McRuer, 2006; Oliver, 2013; Goodley and Lawthom, 2019). These narratives are often situated in medicalised understandings of ideal types, a topic that has been the subject of much criticism in critical disability studies (Kittay, 2001; Berlant, 2007; Kafer, 2021). These narratives place disability as the antithesis of ability, with this bringing with it an understanding of ‘lack’. Outlining ‘crip theory’, McRuer (2006) calls for ‘counter-representations’ that challenge these capitalist notions of bodies. McRuer challenges understanding of disability as lacking in favour of celebrating disability, a sentiment echoed in Goodey’s (2016) work on inclusion phobia. Goodey (2016) draws a distinction between alternative norms and abnormal stating ‘there is of course another way of looking at difference, we could celebrate it, as diversity’ (ibid: 55).

Focusing on lived experiences acts as a way to challenge medicalised narratives. For example, in his research about parents of children with learning disabilities, Thomas (2024) reflects on how participants actively challenged deficit understandings of parenting a child with learning disabilities. Working in direct contrast to dominant narratives, counter-narratives are ‘stories that lie in tension with the ones that we are socialised to expect’ (Andrews et al., 2004: 97). Returning to Shuster and Westbrooks’s conceptualisation of ‘joy deficit’, it has been levelled at disability studies that there is a lack of joy in research (Sunderland et al., 2009). This literature review has pointed to deficit-informed understandings of siblinghood and learning disabilities that are commonplace in the field. In the context of this joy deficit in disability studies, research into lived experience that allows space for joy can contribute to narrative repair regarding people with learning disabilities and siblinghood. Narrative portraiture offers insight into lived experience and space for participant stories to be presented, with nuance, in their own words.

### Narrative portraiture

Narrative portraiture is a method of presenting data which centres participant voices with an extended extract presented from the participants’ words. This paper draws on the approach to portraiture outlined by Rodríguez-Dorans (2022) in the book *Narrative Portraits in Qualitative Research.* Rodriguez-Dorans identifies affects, first-person narratives, and ‘close up storytelling’ as core features of narrative portraits with the goal being one of creating ‘a glimpse into the participants lives which is simultaneously deep, succinct and evocative’ (ibid: 13). To do this, participants’ words are brought together to create a portrait that gives insight into their experiences through the frame of the focus of the research project. The portraits can stand on their own with there often being a clear narrative throughout. This approach echoes wider narrative inquiry through considerations of context and of bridging the gap between personal experiences and the cultural factors that inform them (Crouch and McKenzie, 2006).

Rodríguez-Dorans (2022) advocates an adapted version of the Labovian approach to coding (Labov and Waletzky, 1997) in the formulation of narrative portraits, adding on some extra considerations more specifically focused on portraiture. This sees the researcher break down the analysis into different focus points, with these being encouraged as a guide for coding: characters, orientation in time, complicating action, result and evaluation, small stories, special narrative features and the abstract (Labov and Waletzky, 1997; Rodríguez-Dorans, 2022). Characters is interested in the key actors of the narrative, both those explicitly referenced and those who are implicit. Within this focus, we can begin to ask questions about who are the key figures in the story and who are they to the participant. Orientation in time sees the researcher looking for moments in the narrative that place it, both geographically and with respect to time and chronology. Complicating action refers to ‘concrete situations that disrupt the sense of flow in the life of a participant’ (Rodríguez-Dorans, 2022: 28); these can be major life events or more everyday changes that arise in the narrative as points of tension. Result and evaluation is interested in the outcomes of events in the narrative, both with regard to actual events and also from insights participants gain and reflect upon. Small stories is concerned with examples of stories within the wider narrative. These will be moments where participants tell of experiences that have distinct beginning and endpoints. Special narrative features look towards the themes of the narrative, here the coding would be interested in how the narrative speaks to the research focus. Finally, the abstract is concerned with the overall point of the narrative. In this focus, the narrative is approached more holistically looking at what the narrative tells us and what points are being raised often. This focus on coding allows researchers to engage with participant data with the narrative in mind. In doing this, we get a sense of the story of their data which can then be used to inform a narrative portrait.

In creating a narrative portrait, the participant’s words are brought together in a manner that creates a short story that gives insight into the narrative of their interview. This is not to say that all of the points of coding will be relevant to all narratives; instead, some may be more applicable than others. Through using this approach we are encouraged to produce narrative portraits that give an insight, in the participant’s words, into their experiences that is comprehensive and respectful of their story as a whole. For Smyth and McInerney (2013), it is essential to recognise that the portrait is a product of the researcher and their research focus, arguing the process can be understood as ‘using the informant’s own words, all the while being continually mindful of the need to hold onto the essence of what we have isolated as being most prominent’ (Smyth and McInerney, 2013: 17). Rodríguez-Dorans (2022) builds on this noting how through the act of creating a narrative portrait we make clear the argument of the narrative through piecing together extracts from the participant’s transcript in a manner that makes ‘a compelling story that lays the researcher’s argument for the readers to see’ (ibid: 42).

Rodríguez-Dorans (2022) highlights the link between narrative portraiture and counter-narrative research. This is done through recognition of the potential for the approach to centre marginalised experiences. They argue that for many marginalised groups, their identities are ‘relegated to the fringes of significance’ (ibid: 150), with portraiture offering the chance to make these experiences more central. Furthermore, when read alongside the understandings of damaged identities and calls for narrative repair (Lindemann, 2001), not only does a narrative portrait approach offer the chance to bring identities into the centre, but by doing so also offers new understandings driven by lived experiences. Narrative portraiture offers a chance to centre counter-narratives that are nuanced and human by extension. Smyth and McInerney (2013), for example, place portraits as advocacy ethnography with a focus on participant voice allowing for the disruption of the power imbalance that occurs between participant and researcher. For Rodríguez-Dorans (2022), the approach allows for more open dialogue and understanding to be reached between the reader and participants:

*‘Because when we are able to recognise ourselves in the individual, even if it feels far from our own reality, if we open ourselves to understand ‘the other’ in front of us, not as a research participant but as a sentient being, with drives, needs, and desires, we might be able to look at ourselves through their eyes and recognise ourselves in them.’* (ibid: 140)

This extract captures the disruptive potential of the method. Furthermore, when read alongside the context of a joy deficit in disability studies and the need for more affirmative research there is an argument to be made for the counter-narrative potential of this research approach. Within disability studies, narrative portraits offer a chance to explore lived experiences of disabled people. This is seen in Jacobs et al.’s (2020) research into the transition from school to adult services for young people with learning disabilities. They bring together their narrative portraits from a wide set of data, including interviews with family, service providers and observations in order to create narrative portraits for non-verbal participants. In doing this, they make clear the role of the researcher in constructing narrative portraits whilst also giving insight into everyday life as understood through the data. Connor (2008) produces portraits in their work on the intersection of learning disability, race and class with the aim of ‘creating a picture of how participants understand learning disability as part of their lives’ (ibid: 69). Connor uses the phrase ‘portraits in progress’ to capture the more fluid nature of the work produced, moving away from chronological reflections in favour of ‘asynchronous thoughts and memories’ that come together to offer insight into the research focus.

### Methodology of study

This paper draws on an aspect of the research carried out for my PhD thesis. This research explores the experiences of siblings of people with learning disabilities with a focus on gathering holistic narratives of childhood. To do this, narrative interviews were conducted with 14 siblings of people with learning disabilities aged 18–32 years. Participants were asked to bring along a timeline of their childhood and some photographs to further encourage discussion. These interviews followed Rosenthal’s (2007) narrative framework, which sees the interview split into two sections. First, the participant is given the chance to provide a biographical narration in which they are asked to talk through their timeline with minimal researcher input. The second part of the interview followed a more conventional semi-structured approach with the interviewer asking follow-up questions with the intention of generating richer detail on parts of their narrative. Alongside this, there was a series of questions asking participants about their everyday life growing up that followed the theme of *what was the morning before school like in your house? What was dinnertime like growing up?* These questions worked to ensure that the minutiae of everyday family practices were explored in the interviews. Participants often covered these questions during their biographical narration, but having this set worked to make sure each participant discussed their everyday experiences in some form. The interviews were conducted either online or in-person depending on the preference of the participant. They were recorded and transcribed by myself prior to coding.

The addition of timeline mapping and photo elicitation worked to give participants more control of the interview away from the researcher. By bringing things to discuss, participants were able to shape the interview in a way that was distinct from the interviewer’s input (Mannay, 2010). Alongside this, a creative method element to the study acted to try to ensure comfort for participants in interviews that could have potentially been upsetting (Mason and Davies, 2009). The timeline mapping consisted of participants being asked to ‘bring in a timeline of your childhood’, this brief was deliberately loose and allowed a number of interpretations from those who took part. This varied from handwritten notes of key events and dates to in-depth PowerPoints which incorporated the photographs. The timelines worked as interview prompts, with analysis focusing on how participants discussed them. This was chosen in recognition of the ‘long biographical narration’ as something that is not easy to do. The timelines enabled participants to have notes with them and acted to guide their narration, further contributing to the comfortableness of the interview. The photo elicitation acted similarly with the main role being one of encouraging discussion. Family photographs tend to be ones of joyful moments (Kuhn, 2002) and this meant that often the photographs helped provide comfort for the participants and give them a chance to discuss something more upbeat where necessary.

The transcripts were coded narratively, drawing on the Labovian framework (Labov and Waletzky, 1997) for narrative inquiry as outlined by Rodríguez-Dorans (2022). This required the data to be read with a focus on characters, orientation in time, complicating action, result and evaluation, small stories, special narrative features, and the abstract. Not all of these were relevant in each narrative meaning different portraits draw on these in different ways. The thematic analysis that ran alongside informed some of the narrative coding, particularly around special narrative features and the abstract, with common themes being a central focus in the construction of the portrait (Wells, 2011). In crafting the portrait, the participant’s words were not changed, but the researcher brought together discussion points with the aim of giving insight into how they featured over the course of the narrative. Drawing on Rodríguez-Dorans’s (2022) framework for creating narrative portraits, the excerpts are brought together in a manner that aims to create a ‘compelling story’ whilst making clear the arguments of the researcher with regard to the focus of the project.

It is important to recognise the role of the researcher in this project as well as the choice to interview non-disabled siblings. The project was inspired by my own experiences growing up with an autistic sibling with learning disabilities. As outlined by Meltzer and Kramer (2016) research into siblinghood and disability more widely often reproduces deficit narratives centred around outcomes for non-disabled siblings. This informed my decision to interview non-disabled siblings; however, it must be recognised that the viewpoint of the sibling with learning disabilities does not feature in the portrait. Considering research around “disability by association” (Burke, 2010; Scavarda, 2023), the narrative outlined in this study is not presented to replace those of people with learning disabilities; instead, it is offered with the hope of sitting alongside and contributing to narratives of siblinghood and learning disability that are generative and affirmative.

This paper draws on one narrative portrait as a case study to make clear the affirmative potential of narrative portraiture in understanding learning disability in the everyday. Toby’s portrait (pseudonym) was chosen as his narrative is one that highlights how the methodology can lead to more affirmative understandings of learning disability. Toby will be introduced along with his narrative portrait. This will be followed by the discussion in which the portrait will be analysed using the framework outlined in this section. Specific attention will be given to how Toby’s account of his childhood presented an affirmative counter-narrative of siblinghood and learning disability. The analysis will focus on the everyday experiences that the portrait captures, with particular attention paid to the role of the narrative coding framework as outlined above.

### Toby’s narrative portrait

Toby’s narrative portrait focused on his experience of playing video games with his sister. This played a central role in his narrative, with the games console the Nintendo Wii being afforded its own section of his timeline:

*Nintendo Wii Brother obsessed. Bought on day it came out. Cannot remember when but she* [Beth] *got hooked on Wii Bowling. Our main time spent together at home throughout secondary school, will play some games after dinner or something.*

In the interview itself, the Nintendo Wii was returned to throughout, with Toby describing fond memories of time spent playing with his siblings. The narrative portrait covers the role of the games console during childhood and then further into adulthood and the present. During these reflections, Toby touches on the importance of one-to-one time with his sister, his feelings about his parents, and the joy of playing together. To provide context for the portrait, Toby is in his late twenties and lives abroad as a teacher. He has two siblings, Josh, who is the eldest and then Beth, who is the youngest. Toby is from a white, middle-class family based in the North of England.

### Toby’s narrative portrait: “the best 1 on 1 time that I have with Beth is playing Wii sports together”

*There’s a special shout out to the Nintendo Wii there in the middle [of the timeline] because again, that’s a huge part of our lives, with Beth’s obsession with it I think we have like 5 Wii’s at home so if one breaks we get a new one, I took one to uni and it’s come back, we are stockpiling them at home it feels like. We got it on the day it came out because Josh was completely obsessed with it like for months before it came out coming into the bedroom like ‘oh there’s a new trailer for the new Nintendo Wii’ saying ‘we need to get this game and this game’ you know, my parents probably just found it funny how obsessed with it he was and then they got really into it when we brought it but yeah I cannot remember at what point Beth got really into it but she got really into it and she still plays it today and going into our later childhood, me going to University, even now, like the best 1 on 1 time that I have with Beth is playing Wii sports together.*

*I’ll say it like this, I can never criticise my mum and dad for anything because like it’s the toughest thing that they have got to go through, you know the sacrifices and things like that. The thing that, the only thing that frustrates me a little bit and maybe I should bring it up with my mum and dad a little bit more because Beth will happily stay quiet if she does not have to speak and we are always wanting her to speak and if I, if I like facetime them now and something like that and I start talking to Beth, I try to ask her a question and she’ll be really quiet and my mum and dad will fill in for her and they’ll speak over her and that bugs me a bit because I feel like it’s not helping her with her speech but it’s also not helping mine and her connection which is maybe why I talk about the Nintendo Wii so much because that’s me and her in a different room, the doors closed. It’s an opportunity for me and her to actually spend time together where there’s not really that many other opportunities to do that so yeah and she’s, oh man, she’s amazing at it as well. She has this technique with the controller right where she, she has such grace she kind of gets her hand down and flicks it up to the side, the same hand motion and she’ll be like stern her face will have no expression and she’ll flick it up to the side and the ball does the most, the maddest like swerve so she can set up her shot beforehand she goes right to the edge of the lane as far as you can go and she does this swerve and honestly 9 times out of 10 it’s a strike, like every time. I remember having, we are at my Auntie’s house having christmas dinner and they had like, the dining room has a mirror looking into the conservatory and Beth has taken herself off to play a bit of bowling, the adults and some of the kids are chatting, and I could see her through the window, I was having a little look like ‘nice strike’ and then we keep chatting and then I have another look and she has got like 8 strikes in a row like that and she’s just slinging them out but yeah so that is a big part of our enjoyment together and the sort of stuff that we did.*

## Discussion

Toby’s portrait touches on a number of aspects of his sibling relationship, with the central theme being the role played by the Nintendo Wii games console growing up. The portrait will be unpacked with regard to the narrative focus points outlined by Rodríguez-Dorans (2022), these being characters, orientation in time, complicating action, result and evaluation, small stories, special narrative features, and the abstract. Whilst these provide a framework for analysis, specific attention will be given to the more affirmative nature of the portrait throughout the analysis in order to ensure the research aims are met.

### Characters

In creating the narrative portrait, it was important to ensure that the characters central to Toby’s narrative were represented in a manner that reflected their importance to his story. Recognising the limitations of a portrait’s size alongside the focus of the research project, the result was a portrait that centred around family. The first character to mention is Toby as the narrator, throughout he references his feelings about certain events firmly placing himself within the narrative he is telling. Alongside Toby as the narrator, his sister Beth is the central character of the portrait. The stories focus on Toby’s relationship with Beth and how this has shifted over time, with the role of playing video games together being a consistent thread in the story. Wider family also feature in the portrait. For example, Josh, their brother, is mentioned at the start as the reason the family got their Nintendo Wii. Toby’s parents are then brought into the story initially to get the Nintendo Wii and getting ‘really into it’ alongside the children. As the portrait progresses, we see Toby discussing his relationship with Beth, and the role his parents play in that over time. The final section sees Toby recount a funny story of a family Christmas in which a number of characters are referenced, both explicitly (their Aunt) and implicitly as being at this Christmas dinner.

### Orientation in time

The orientation in time of a narrative refers to a number of things alongside temporality. With regard to this portrait, significance is also found in the geography of the story and the everyday as a setting. Beginning with the context of the portrait, this is found at the beginning with Toby reflecting on getting a Nintendo Wii on the release date, which places Toby’s childhood, and the beginning of the portrait in the mid-2000s. This is not the only timeframe in which the extract takes place, the present day becomes the focus as we move into the discussion around calling his family from abroad. This aspect also places the portrait geographically, with Toby juxtaposing the time spent together at home as children with his present day awareness of the importance of putting work into his relationship with his sister and his parents’ role in this. This is with regard to Toby now living abroad and not seeing his family as much, a context that is implied in his reflections. Whilst this can be seen in the central geographical orientation of the portrait, we also see more at-home reflections on space, for example, Toby’s feelings about being in a different room and this privacy affording a certain kind of catch-up with his sister that he is very fond of.

Alongside the temporal and spatial orientations of Toby’s narrative, we also see the portrait play out firmly in the everyday. The stories and reflections are ones about Toby’s everyday life and more, arguably, mundane experiences. This, of course, relates to the focus of the research project, with this being an aim that is considered whilst bringing the portrait together. Furthermore, considering the affirmative potential of narrative portraits this setting recognises the more joyful and humorous aspects of Toby’s narrative as part of his everyday family life. Toby’s account makes clear the everyday joy of living alongside his sibling. Davies (2023) refers to ‘living alongside’ as a key element to what makes siblinghood unique arguing ‘the idea that, even if sometimes too close for comfort, the heightened proximities of sharing a home with one’s sibling(s) afford a particular kind of ‘living alongside’ is important’ (ibid: 96). Davies’ reflection acknowledges the ups and downs of siblinghood in the everyday, with the ‘heightened proximity’ bringing with it potential for frustrations alongside closeness. From Toby’s portrait, we see how this living alongside can be characterised by fun through things such as playing together. This playing together is something that characterises Toby’s relationship with Beth, and more widely his family’s relationships with their brother and parents being involved. The centrality of everyday joy to this portrait is important when considered within the context of joy deficit within disability studies (Sunderland et al., 2009; Shuster and Westbrook, 2022) as it can be seen to actively reject deficit understandings of living with a sibling with learning disabilities.

### Complicating action

For Patterson (2013), complicating action coding works to ‘relate the events of the story and typically follow a ‘then, and then’ structure which gives a linear representation of time and permits an open-ended series of events to be related’ (ibid: 31). Within the portrait, we see a number of these moments. Starting with the family acquiring their Nintendo Wii which becomes the central focus of the portrait. The joy and fun that characterised Toby’s narrative are captured in these moments, with the portrait using the game console as the foundation by which this aspect of the narrative is made clear. The Nintendo Wii is central in all reflections in the portrait, even as we move into the next complicating action of Toby moving away and keeping in contact with his family. We see this time spent playing the Nintendo Wii as almost his ‘gold standard’ of hanging out with his sister which he uses as a comparison point for more difficult interactions such as facetime with his family. This context makes clear why playing the Nintendo Wii with his sister is important to Toby as it helps to facilitate this 1 on 1 time. This time is essential in Toby’s view to keep his connection to Beth, especially now he does not live at home. Considering the relational nature of siblinghood (Davies, 2023) this activity is presented as a core way Toby and Beth enact their sibling relationship. The fact Toby puts in such effort to keep this connection and is frustrated when he feels his parents unintentionally block this, once again pushes back against a deficit understanding of siblinghood and learning disability. Whilst in one sense this is a very everyday portrait of sibling gaming and hanging out, it takes on a new meaning when considered alongside commonplace narratives of deficit (Meltzer and Kramer, 2016). Viewed alongside the notion of a ‘joy deficit’ Toby’s portrait offers a story of inclusion, love, and fun that pushes an affirmative narrative.

### Result and evaluation

The result and evaluation sections of the narrative link closely to the complicating action. For Riessman (1993) evaluation is ‘the soul of the narrative’ (ibid: 21). Patterson (2013) equally captures the importance of this aspect arguing it ‘mediates the crucial ‘point’ of the story, thereby justifying its telling, and it reveals the narrator’s perspective on the events being told’ (ibid: 31). In Toby’s portrait, we can trace the result and evaluation alongside the complicating actions. Starting with acquiring the Nintendo Wii games console, we see the result being this special time that Toby and his family spend playing together and the closeness this brings, even now in the present day. Toby’s evaluation of this is one of closeness and appreciation for this time together alongside admiration for his sister’s talent at the game. The joy that runs through the portrait is drawn from this fun that characterised their childhoods, and the present as shown when Toby sees his family in person. The reflection that it is ‘the best 1 on 1 time that I have with Beth’ makes clear the importance Toby places on this time.

The result and evaluation that come with Toby’s reflection on moving away is found in his feelings about calling home and speaking to Beth and his parents. The result of the action of moving away is calling home and not getting the chance to speak directly to Beth, or feeling as though this is not the quality time that he was having at home with her. In the evaluation of this Toby is both considerate of his parents’ experience whilst also making clear his own frustrations around the situation. Toby’s reflection on his parents touches on feelings of sacrifice and an understanding from Toby of the challenges people with learning disabilities and their families face in the UK (Goodley et al., 2014; Ryan, 2019). It is interesting how Toby does not feel as though he can really criticise his parents for this reason even though his frustrations lie with their role in his relationship with his sister. This feeling reflects wider research around siblings of people with learning disabilities and structural factors that influence their childhoods. Toby’s notion of sacrifice is rooted in the structural ableism that families of people with learning disabilities have to navigate throughout their lives (Goodley, 2014). Furthermore, whilst the want for his sister to talk more in the calls is partly reinforcing developmental narratives that are important to call out (Gabriel, 2021), there is also an element of care to this standpoint, both for his sister but also in the understanding he shows for his parents.

### Small stories

Small stories refer to the sections of the narrative where the participant recounts a story from beginning to end that could be read on its own, for Rodríguez-Dorans (2022) the key to a small story is the plot, and the ‘potential to be analysed as units of meaning’ (ibid: 31). Whilst not all portraits will have a small story embedded due to the size limitations of most research outputs, Toby’s portrait includes the story of the family Christmas dinner. This story has a distinct beginning and end, with the story starting with the discussion of Beth’s bowling technique and finishing with the reflection ‘so that is a big part of our enjoyment together and the sort of stuff that we did’. The story can be analysed for its content as an individual story, but also as part of the wider narrative. The story is funny, with Toby juxtaposing the nature of Wii bowling with his sister’s approach to playing to create a funny image of focus. This evokes a sense of banter between the siblings, something that was apparent more widely in his narrative. Furthermore, where this could be read as Toby making fun of his sister, this humorous framing is combined with genuine admiration for how good Beth is at the game. These factors come together to give an insight into Toby’s sibling relationship that is built on humour and care echoing Lampert and Ervin-Tripp’s (2006) reflection on teasing as requiring closeness and understanding.

### Special narrative features

Special narrative features refer to the themes of the narrative and give an overview of what could be potentially important to focus on in a narrative portrait. Toby’s portrait is centred around the core themes of his narrative: humour, closeness, and joy. By focusing on gaming with his sister, these themes are each afforded space within the portrait whilst also having a narrative thread that ties the extract together. Furthermore, the central aim of a narrative portrait of speaking to the research focus (Rodríguez-Dorans, 2022) is met with the portrait giving an insight into affirmative reflections on siblinghood and learning disability. The portrait makes clear Toby’s sense of humour. This is seen at the beginning with the reflection on how the family came to get their Nintendo Wii with Toby poking fun at his brother for how obsessed with it he was. For Lampert and Ervin-Tripp (2006), teasing is considered a sign of closeness as it requires an understanding from both parties so as to not be interpreted as an insult. Furthermore, Davie’s (2023) reflections on ‘living alongside’ refer to a heightened proximity which brings with it space for closeness but also at points more conflictual teasing. Whilst Toby’s narrative portrait features humour and teasing that is quite ‘light’ this approach to sibling teasing offers an opportunity to analyse joking in a manner that is understanding. The humour of Toby’s portrait is linked clearly to the affirmative nature of the piece, with his approach to storytelling contributing to a portrait that is, in most places, fun and centred around the joy of their childhoods.

Closeness is the second theme that came through in Toby’s narrative and is apparent within the portrait also. The overall piece points to the family being close, with a specific focus on Toby’s relationship with Beth. The most obvious reference to this is in the section around moving away and maintaining closeness, with Toby reflecting on the shared experience of playing video games as a central part of their sibling relationship. Whilst the discussion is centred around the challenges of maintaining a relationship whilst living away from home, we also get a sense of how important fun is to Toby and Beth, as shown in the reflection ‘it’s also not helping mine and her connection which is maybe why I talk about the Nintendo Wii so much because that’s me and her in a different room’. Here, Toby makes clear why he spoke about gaming so much in his narrative as it brings with it one-to-one time to spend with his sister that they both really value. His want to ‘help’ their connection is driven by his awareness that it is harder to have this 1-on-1 time when he is not at home and therefore he feels some concern about how they will maintain their closeness.

The final special narrative feature from the narrative that was incorporated into the portrait is joy with Toby’s portrait being one that highlights the fun of their childhood and even in the present day how having fun is one of the central ways he and Beth maintain their relationship. The portrait is framed around fun and the joy that comes with that, with Toby’s narrative presenting an understanding of siblinghood and learning disability that places joy at its centre. Toby was keen in his narrative to ensure he captured the fun that he feels is a core part of his sibling relationship as further highlighted by the section within the timeline dedicated to the Nintendo Wii. All of the special narrative features come together to give an insight into the everyday of Toby’s childhood, and the fun of playing games with his family. In this regard, the portrait works to counter deficit understandings of siblinghood and learning disability. Taking into account the joy deficit that can be argued to exist within disability studies (Sunderland et al., 2009), Toby’s narrative, and the portrait created from it, ensure joy is presented as a prerequisite for understanding Beth and his relationship.

### The abstract

The abstract attempts to capture the overall point of the narrative, answering the question: what is it telling us? This information is then used in bringing together the portrait, giving a basis to guide the researcher around what is important to include. Toby’s narrative captures the nuance of everyday siblinghood (Davies, 2023), whilst centring joy and humour in particular. In bringing together the portrait, the recurring references to the Nintendo Wii felt like the perfect place to focus the piece as it allowed both reflections on fun and joy but also gave space for Toby’s reflections on closeness and having moved away. Narrative portraiture offers an insight that allows for the nuance of everyday sibling relationships to come through. Toby’s feelings are contextualised amongst his wider narrative to ensure that his words are given appropriate space for the reader to understand his experiences. For example, the reflections around his parents and calling home are tied up in wider feelings about keeping a connection to his sister. Similarly, the joking about his sister’s bowling skills is read alongside the admiration and joy he feels when gaming with her. These factors come together to give an overview of Toby’s narrative that centres joy, closeness, and humour in an affirmative way.

## Conclusion

Narrative portraits offer one avenue through which to address the joy deficit in disability and siblinghood research. By giving insight into participants’ narratives, in their own words, the nuances of living with siblings are captured in a way that does not inadvertently contribute to commonplace narratives of deficit. Reflecting on their research with parents of disabled children, Thomas (2024) argues ‘parents revolt against dominant conceptions of parenting a disabled child as a source of despair, fear, and no future’ (ibid: 2). The same can be said from a sibling perspective for Toby, with his narrative portrait actively rejecting a deficit understanding of his sibling relationship in favour of one that places joy and fun as key parts of the relationship. Goodley (2023) calls for the centring of the human in understandings of learning disability, something that we can see in Toby’s narrative portrait. Toby’s portrait presents his experience as a sibling in a nuanced light, where challenges can be acknowledged but in a manner that avoids playing into commonplace deficit narratives of disability and siblinghood. For Chapman (2005) portraiture ‘depicts the multiple layers of contexts represented by events and people’ (ibid: 28), this understanding makes clear how the context a portrait affords lends itself to nuanced presentations of lived experience. Furthermore, throughout the portrait we see the care the siblings feel for each other, captured in Toby’s concerns around maintaining the relationship after moving away. This nuance is achieved through the space extended to Toby’s narrative that allows for an overview that can highlight different aspects of his experience in his own words. For Smyth and McInerney (2013), this is a political choice, with there being a sense of accessibility to the method in how it allows a clear picture of the participants’ experience to be presented to the reader. They build on this, arguing ‘as researchers we have a moral and ethical responsibility beyond the ‘thin’ imposed views of university ethics committees—to work with and advance the lives of those who are institutionally and systematically the most excluded and silenced’ (ibid: 17). Acknowledging this, it is important to note the need for more research that explores the experiences of people with learning disabilities as siblings, an argument that has been made by Richardson and Jordan (2017). Whilst Toby’s portrait can be seen to contribute to narrative repair regarding understandings of siblinghood and learning disability through the affirmative narrative it offers, it is not the complete picture, and therefore more research that centres people with learning disabilities’ sibling experience is needed.

## Data Availability

The original contributions presented in the study are included in the article/supplementary material, further inquiries can be directed to the corresponding author.
